# Cardioprotective Actions of the Annexin-A1 N-Terminal Peptide, Ac_2-26_, Against Myocardial Infarction

**DOI:** 10.3389/fphar.2019.00269

**Published:** 2019-04-03

**Authors:** Cheng Xue Qin, Sarah Rosli, Minh Deo, Nga Cao, Jesse Walsh, Mitchel Tate, Amy E. Alexander, Daniel Donner, Duncan Horlock, Renming Li, Helen Kiriazis, Man K. S. Lee, Jane E. Bourke, Yuan Yang, Andrew J. Murphy, Xiao-Jun Du, Xiao Ming Gao, Rebecca H. Ritchie

**Affiliations:** ^1^Baker Heart and Diabetes Institute, Melbourne, VIC, Australia; ^2^Department of Pharmacology and Therapeutics, The University of Melbourne, Parkville, VIC, Australia; ^3^Department of Diabetes, Central Clinical School, Monash University, Melbourne, VIC, Australia; ^4^Department of Pharmacology, Monash University, Clayton, VIC, Australia; ^5^Centre for Inflammatory Diseases, Monash University, Clayton, VIC, Australia

**Keywords:** myocardial ischemia, inflammation, cardiac remodeling, annexin-A1, formyl peptide receptors

## Abstract

The anti-inflammatory, pro-resolving annexin-A1 protein acts as an endogenous brake against exaggerated cardiac necrosis, inflammation, and fibrosis following myocardial infarction (MI) *in vivo*. Little is known, however, regarding the cardioprotective actions of the N-terminal-derived peptide of annexin A1, Ac_2-26_, particularly beyond its anti-necrotic actions in the first few hours after an ischemic insult. In this study, we tested the hypothesis that exogenous Ac_2-26_ limits cardiac injury *in vitro* and *in vivo.* Firstly, we demonstrated that Ac_2-26_ limits cardiomyocyte death both *in vitro* and in mice subjected to ischemia-reperfusion (I-R) injury *in vivo* (Ac_2-26,_ 1 mg/kg, i.v. just prior to post-ischemic reperfusion). Further, Ac_2-26_ (1 mg/kg i.v.) reduced cardiac inflammation (after 48 h reperfusion), as well as both cardiac fibrosis and apoptosis (after 7-days reperfusion). Lastly, we investigated whether Ac_2-26_ preserved cardiac function after MI. Ac_2-26_ (1 mg/kg/day s.c., osmotic pump) delayed early cardiac dysfunction 1 week post MI, but elicited no further improvement 4 weeks after MI. Taken together, our data demonstrate the first evidence that Ac_2-26_ not only preserves cardiomyocyte survival *in vitro*, but also offers cardioprotection beyond the first few hours after an ischemic insult *in vivo*. Annexin-A1 mimetics thus represent a potential new therapy to improve cardiac outcomes after MI.

## Introduction

Myocardial infarction (MI or ‘heart attack”) and its resultant heart failure (HF) remains a major cause of death and disability, despite current thrombolytic and interventional coronary revascularization procedures (Hausenloy et al., 2013; Heusch, 2013). Development of novel strategies is essential to overcome the limitations of these approaches and the increasing morbidity and mortality due to MI. Given that MI represents a severe inflammatory insult to the myocardium, pro-resolving mediators may represent an exciting therapeutic target in this regard.

The therapeutic potential of the glucocorticoid-regulated anti-inflammatory mediator, annexin-A1 (ANX-A1), has been recognized in a range of systemic inflammatory disorders (Sugimoto et al., 2016; Ansari et al., 2018). ANX-A1 belongs to the annexin superfamily, which has ≥ 13 protein members; these differ most commonly only at their N-terminal tails (Flower and Blackwell, 1979; Gavins et al., 2003a). The N-terminal annexin-A1 peptide Ac_2-26_ was initially reported to elicit similar biological effects (such as inhibition of neutrophil adhesion and infiltration) as the full ANX-A1 protein, suggesting that at least part of the anti-inflammatory actions of ANX-A1 can result from its N-terminal proteolytic cleavage products (Harris et al., 1995; La et al., 2001a,b). Further, both ANX-A1 and Ac_2-26_ exhibit pro-resolving properties in several animal models, such as a pleurisy model of acute inflammation (Corminboeuf and Leroy, 2015; Perretti et al., 2015; Lima et al., 2017).

ANX-A1 and its mimetic peptide, Ac_2-26_ bind to the formyl peptide receptor (FPR) family of 7 transmembrane G-protein coupled receptors (GPCR), to inhibit neutrophil activation, migration and infiltration (Perretti and Flower, 1995; Perretti et al., 1995; Chatterjee et al., 2005; Dalli et al., 2008). There are three FPR subtypes in humans, FPR1, FPR2, and FPR3 (Ye et al., 2009; Qin et al., 2015). Encoded by the *FPR1* gene, FPR1 is predominately located on neutrophils, epithelial cells and endothelial cells in multiple organs (Becker et al., 1998). Although it has a similar distribution pattern to FPR1, FPR2 has largely been considered to mediate the majority of the anti-inflammatory effects of ANX-A1 (Gavins et al., 2003b, 2005; Ye et al., 2009; Dufton et al., 2010; Maderna et al., 2010). For example, ANX-A1 fails to inhibit migration of macrophages isolated from FPR2 knockout mice *in vitro* (Dufton et al., 2010), suggesting that this anti-inflammatory action of ANX-A1 is mediated by FPR2 (Dufton and Perretti, 2010). In contrast to FPR1 and FPR2, FPR3 are highly expressed on dendritic cells and monocytes, with much lower levels detected in heart, and less information regarding the role of FPR3 is presently available (Ye et al., 2009). Interestingly, it has been suggested that Anx-A1 and Ac_2-26_ could promote an anti-inflammatory and pro-resolving signature, via interleukin (IL)-10 release secondary to induction of FPR1/2 heterodimers (Cooray et al., 2013).

Several lines of evidence indicate that endogenous ANX-A1 protects the myocardium from acute ischemic injury (Ansari et al., 2018). In isolated mouse hearts, deficiency of ANX-A1 further impairs recovery of left ventricular (LV) function, and markedly reduces the activity of the pro-cell survival kinase, Akt, following ischemia-reperfusion (I-R) *in vitro* (Qin et al., 2013). Excitingly, we have recently revealed the importance of endogenous ANX-A1 in the setting of I-R injury (Qin et al., 2017a). Our evidence suggests ANX-A1 is likely acting as an anti-inflammatory and pro-resolving meditator, dampening both I-R induced inflammation and myocardial damage. Mice deficient in ANX-A1 exhibited increased LV necrosis, inflammation and adverse remodeling compared with wildtype mice, after myocardial ischemic insult *in vivo* (Qin et al., 2017a). Given that macrophages and neutrophils invade myocardial tissue in this setting and contribute to injury, activation of FPRs may be a novel pharmacological intervention for treating I-R injury.

The protective effects of ANX-A1 in the cardiovascular system are further supported by evidence obtained using exogenous ANX-A1 and Ac_2-26_ *in vitro* and *in vivo* (Qin et al., 2015). ANX-A1 has been shown to attenuate neutrophil recruitment and adhesion to endothelial cells (Dalli et al., 2008). In addition, Ac_2-26_ has also been demonstrated to elicit direct cardioprotective actions (i.e., in the absence of circulating neutrophils and other pro-inflammatory cells). These include preservation of cardiac contractile function and cardiomyocyte viability, in both isolated cardiomyocytes obtained from the post I-R rat hearts and other settings of myocardial stress *in vitro* (Ritchie et al., 1999, 2003, 2005; Qin et al., 2013). The anti-inflammatory actions of ANX-A1 and Ac_2-26_ have also been investigated in short-term (<2h) models of I-R *in vivo*. The anti-inflammatory actions of ANX-A1 are evidenced by reductions in infarct size and reduced neutrophil infiltration (Gavins et al., 2003a; La et al., 2001a,b). To date, the majority of studies which have examined the cardioprotective roles of exogenous ANX-A1 have, however, utilized relatively short periods of ischemia and reperfusion (<2 h). Thus, elucidating the cardioprotective potential of the ANX-A1 mimetic, Ac_2-26,_ over longer periods of ischemia, and at later timepoints following reperfusion *in vivo*, *is* clearly warranted. This would address the translational potential and clinical relevance of targeted ANX-A1 therapy in the management of ischemic heart disease, in both the immediate (24 h) and longer-term.

## Materials and Methods

### Animals and Materials

All animal research was conducted in accordance with the National Health and Medical Research of Australian Code and Practice and use of animals for scientific purposes, and the study and protocol were reviewed and approved by the Alfred Medical Education Precinct Animal Ethics Committee (E/1154/2011/B), and Directive 2010/63/EU of the European Parliament in the protection of animals used for scientific purpose. Neonatal (1–2 days old) Sprague-Dawley rats (mixed sex) and male C57/BL6 mice (11–14 weeks-of-age) were bred and housed in the Alfred Medical Research and Education Precinct (AMREP) Animal Centre and maintained under a 12 h light/dark cycle. All adult animal experiments were reported in the form of the “Animal Research Reporting of *in vivo* experiments (ARRIVE) Guidelines” (Drucker, 2016), as outlined in Supplementary Figure S1.

All reagents were purchased from Sigma-Aldrich (St Louis, MO, United States) except where indicated, and were of analytical grade or higher. Dulbecco’s-modified Eagle Medium (DMEM) and fetal bovine serum (FBS) were purchased from Gibco (Gaithersburg, MD, United States) and JRH Biosciences (Lenexa, KS, United States). All materials used for cardiomyocyte isolation were of tissue culture grade. Ac_2-26_ was synthesized by Chemieliva (Chongqing, China).

### Isolation and Culture of Primary Ventricular Cardiomyocytes and Cardiofibroblasts

Cardiomyocyte isolation from neonatal rats was performed by serial enzymatic digestion, as previously described (Irvine et al., 2012, 2013; Qin et al., 2017b). Cardiomyocytes were suspended in sterile DMEM, supplemented with 100 U/mL penicillin, 100 μg/mL streptomycin, and 10% FCS. The myocyte-rich cell suspension was pre-plated twice (45 min at 37°C) to reduce fibroblast contamination. Cardiomyocytes were then plated on 60-mm dishes for gene expression analysis, and on 12-well plates at 1.3 × 10^5^ cells per cm^2^ to assess cardiomyocyte injury, in the presence of 1% 5-bromo-2′-deoxyuridine (BrdU, to limit proliferation of any remaining fibroblasts) (Qin et al., 2017b). Neonatal mouse cardiac fibroblasts were obtained as a by-product of cardiomyocyte isolation (Irvine et al., 2012). Cardiofibroblasts (passage #3) were seeded on 60-mm tissue culture dishes at 9.5 × 10^4^ cells per cm^2^ and allowed to grow to 80% confluence before study.

### RNA Extraction and Quantitative-Real Time PCR (qPCR)

RNA was extracted from cardiomyocytes, cardiac fibroblasts or LV tissues using TRIzol^®^ (Invitrogen, Life Technologies, Mulgrave, VIC, Australia) as previously described (Huynh et al., 2012). Briefly, tissue were homogenized using sterilized zirconium oxide beads in TRIzol^®^ with a tissue lyser (Tissue Lyser II Qiagen^®^). Homogenates were then centrifuged and chloroform was added to separate the aqueous and organic layers. RNA in the aqueous layer was precipitated in isopropanol overnight at -20°C. The RNA pellets were washed in ethanol and resuspended in RNAase-free water. RNA quality was assessed by Nanodrop2000 spectrophotometer (Thermo Fisher Scientific; wavelength 260 nm) and treated with DNAse treated using commercial Ambion DNase I Kit (Ambion^®^; Thermo Fisher Scientific) according to the manufacturer’s instructions. Taqman reverse-transcription reagents (Applied Biosystems, Mulgrave, VIC, Australia) were used to generate approximately 20 ng/μl cDNA from 1 μg of DNase-treated RNA via transcription. Quantitative-real time PCR (qPCR) was conducted using SYBR green chemistry (Life Technologies, Victoria, Australia) to measure the expression of genes of interest using the Applied Biosystems ABI Prism 7700 Sequence Detection System described previously using sequences in Table 1 (Qin et al., 2017a). The comparative 2^-ΔΔCt^ method was used to analyze changes in gene expression as a fold change relative to sham mice, with ribosomal 18S as the housekeeping gene (Qin et al., 2017a). If cycle threshold (Ct) for the gene of interested yield a result of “undetermined.” i.e., Ct > 40, expression was reported as zero (i.e., not detected).

**Table 1:** 5′-3′ primer sequences used for gene expression by qPCR.

	Forward primer	Reverse primer
*18S*	TGT TCA CCA TGA GGC TGA GAT C	TGG TTG CCT GGG AAA ATC C
*rFpr1*	CAC CTC CAC TTT GCC ATT TT	TGC ACA TGA ACC AAC CAA AT
*rFpr2*	GCT CAG AAC CAC CGC ACT	CAT AAA TCC AGG GCC CAA C
*rFpf3*	TGT TCA CCA TGA GGC TGA GAT C	TGG TTG CCT GGG AAA ATC
*mFpr1*	CCT TGG CTT TCT TCA ACA GC	GCC CGT TCT TTA CAT TGC AT
*mFpr2*	ACA GCA GTT GTG GCT TCCTT	CCT GGC CCA TGA AAA CAT AG
*mCtgf*	TGA CCC CTG CGA CCC ACA	TAC ACC GAC CCA CCG AAG ACA CAG
*mTgf*-β	TGG AGC AAC ATG TGG AAC TC	GTC AGC AGC CGG TTA CCA
*mSerca-2a*	TCT GGA GTT TTC ACG GGA TAG AA	TGT CCG GCT TGG CTT GTT
*mmyh7*	TCT CCT GCT GTT TCC TTA CTT GCT A	GTA CTC CTC TGC TGA GGC TTC CT
*mCd68*	CCA ATT CAG GGT GGA AGA AA	CTC GGG CTC TGA TGT AGG TC
*mS100a8*	CCA TGC CCT CTA CAA GAA TGA	TAT CAC CAT CGC AAG GAA CTC
*mS100a9*	TCA TCG ACA CCT TCC ATC AA	GTC CAG GTC CTC CAT GAT GT
*mArg-1*	TCA GAA GCT GTT CTT GGT CTG AAC	GTT CAT GGG GAT CCC AGT GA
*mCd206*	GGC TAC CAG GAA GTC CAT CA	TGT AGC AGT GGC CTG CAT AG

### FPR Expression in Cardiac Cells *in vitro*

The optimal concentrations of Ac_2-26_ (over 0.3–3 μM) in hypoxic cardiomyocytes was determined initially in pilot studies (see Supplementary Table S1); 1 μM was demonstrated as the optimum concentration and was used in all subsequent cardiomyocyte studies. Cardiomyocytes plated on 60-mm dishes were incubated for 48 h at 37°C in serum-free DMEM, in the presence or absence of Ac_2-26_ (1 μM) or vehicle (0.1% DMSO). At the end of 48 h, RNA was extracted and qPCR performed) to determine the relative gene expression of rat Fpr subtypes (*rFpr1, rFpr2, and rFpr3*, primer sequences detailed in Table 1) relative to the housekeeping gene 18S, as described previously (Qin et al., 2017b). Cardiac fibroblasts were starved overnight in serum-free DMEM, before incubation for 24 h at 37°C with Ac_2-26_ (10 μM) or vehicle, prior to RNA extraction for qPCR determination of the relative gene expression of *mFpr1* and *mFpr2* (refer to primer sequences in Table 1), again relative to the housekeeping gene 18S, as described previously (Qin et al., 2017b).

### Cardiomyocyte Injury Responses *in vitro*

Following 48 h in serum-free DMEM, cardiomyocytes plated on 12-well tissue plates were subjected to simulated ischemia, induced by replacement of culture media with sterile-filtered Krebs buffer (118 mM NaCl, 4.8 mM KCl, 1.2 mM KH_2_PO_4_, 1.2 mM MgSO_4_, 25 mM NaHCO_3_, 50 mM EDTA. 11.0 mM glucose, 1.75 mM CaCl_2_), before incubation for 6 h at 37°C under hypoxia (95% N_2_-5% CO_2,_ using a hypoxia chamber, QNA International, Melbourne, VIC, Australia), with subsequent 48 h reoxygenation as described previously (Qin et al., 2017b). Agonists were dissolved in DMEM or Krebs buffer, and were present for the full duration of hypoxia (H) and reoxygenation (R). At the end of 48 h reoxygenation, culture supernatant aliquots were collected on ice and stored at -80°C for assessment of cardiomyocyte injury via levels of lactate dehydrogenase (LDH) released (Cyto-Tox-One^TM^ Homogeneous Membrane Integrity Assay, Promega Inc, Dane County, WI, United States). Levels of cardiac troponin (cTnI) released from cardiomyocytes were also determined using a high-sensitivity rat cTnI ELISA kit (Life Diagnostics Inc, West Chester, PA, United States) as per manufacturer’s instructions.

### Cardiac Injury Responses *in vivo*

Adult male C57BL/6 mice (22–30 g) were randomly assigned to either myocardial I-R injury (cohort 1–3), permanent coronary artery ligation (cohort 4) or sham *in vivo* (Supplementary Figure S1). Briefly, mice were anesthetized, ketamine/xylazine/atropine (KXA, 100/20/1.2 mg/kg, i.p.), mechanically ventilated and a left thoracotomy performed to expose the left anterior descending (LAD) coronary artery, as previously described (Gao et al., 2005, 2010, 2011). For Cohorts 1, 2, and 3, the LAD was ligated using 7.0 silk suture with a slip-knot enclosing two releasing rings. Regional ischemia was induced for 40–60 min, then blood flow through the LAD was re-established by releasing the slip-knot, as we have described previously (Gao et al., 2005, 2010, 2011). Mice in cohorts 1–3 were then subjected to reperfusion for 24, 48 h, and 7-days respectively, as these are considered optimal timepoints for the assessment of cardiac necrosis, inflammation and early remodeling *in vivo* respectively, as reported previously (Qin et al., 2017b). Sham animals underwent identical surgical procedures, but without ligation. Mice were randomly assigned to receive either vehicle (10% dimethyl sulfoxide, DMSO) in saline, i.v.) or Ac_2-26_ peptide (1 mg/kg, i.v.) every 24 h, for cohorts 1–3, with the first dose administered immediately before reperfusion (until day 7).

An additional cohort of mice, cohort 4, was subjected to a more severe ischemic insult, permanent LAD ligation; late cardiac remodeling and cardiac dysfunction were assessed in these mice after 4 weeks in sham and MI mice ± vehicle (10% DMSO in saline, s.c., osmotic pump) or Ac_2-26_ (1 mg/kg/day, s.c., osmotic pump) from the time of cardiac surgery until experimental endpoint. The initial osmotic pumps (Alzet, Model 1002, Cupertino, CA, United States) were implanted under anesthesia at the time of LAD occlusion surgery. Two weeks later, the first pumps were removed and a second pump placement was performed under isoflurane anesthesia (induction at 3–4%, followed by 1–2% isoflurane, to maintain anesthesia during surgery). For all cohorts, infarcted or sham-operated mice were killed under KXA anesthesia at the end of study, and heparinised blood collected by cardiac puncture for later analysis as indicated.

### Assessment of Cardiac Necrosis *in vivo*

Plasma levels of cTnI were determined in cohort 1 (following 40 min ischemia and 24 h reperfusion) using ELISA as for cardiac myocytes. To further evaluate the extent of cardiac necrosis, infarct size (IS) in relation to the area-at-risk (AAR) was also determined, using 2,3,5-triphenyltetrazolium chloride (TTC) staining of cardiac sections, as described previously (Qin et al., 2017a,b). The images were analyzed in a blinded fashion using Image J (Version 1.45S, National Institute of Health, United States). The non-ischemic zone (blue area), area-at-risk zone (AAR, red and white-yellow areas), infarct zone (white-yellow areas) and total LV were outlined, and infarct size was calculated as the percentage of the infarct zone/AAR zone (Qin et al., 2017a,b).

### Assessment of Cardiac Inflammation *in vivo*

Lung, atria, left and right ventricles from mice in cohort 2 (following 60 min ischemia and 48 h reperfusion) were dissected, blotted dry and weighed. LVs were cut in two at the occlusion site, and the apical half placed in Tissue-Tek optimal cutting temperature compound (OTC, Tissue-Tek, Torrance, CA, United States) for storage at -80°C. The apical half of LVs were sectioned at 6 μm for immunofluorescent analysis. Sections were pre-incubated with 4% paraformaldehyde for 20 min, and 10% normal goat serum (NGS) for a further 30 min. Sections were then incubated at room temperature with either CD68^+^ or Ly-6B.2^+^ primary antibody (1:200, ABD Serotec, Raleigh, NC, United States) for 1 h, followed by 30 min incubation with the Alexa Fluor 546 secondary antibody (1:200, Invitrogen, Carlsbad, CA, United States), to detect cardiac inflammatory cells infiltration, respectively. Finally, sections were incubated with 0.001% Hoechst 33342 (Invitrogen, Melbourne, VIC, Australia) for 30 min, to elicit nuclear staining. Images were photographed and analyzed as described previously (Qin et al., 2017b). For detection of neutrophil infiltration into the heart, sections were incubated overnight at 4°C with Ly-6G^+^ primary antibody (1:400 in 5% NGS and 0.01% PBS-T, BD Pharm, Scoresby, Australia), followed by incubation at room temperature for 2 h with Alexa Fluor 546 secondary antibody (1:1000 in 5% NGS and 0.01% PBS-T, Invitrogen, Carlsbad, CA, United States). Finally, sections were incubated with 1 mg/ml 4′,6-diamidino-2-phenyldole (DAPI, 1:1000 in PBS) for 10 min and quenched with Sudan Black (0.1% in 70% ethanol), to elicit nuclear staining and reduce auto-fluorescence, respectively. Images were photographed and analyzed as described previously (Qin et al., 2017b).

Left ventricular tissues collected from mice in cohort 3 (following 40 min ischemia and 7-days reperfusion) were fixed in neutral-buffered formalin, embedded in paraffin (Alfred Pathology Service, Melbourne, VIC, Australia) and then sectioned at 4 μm. Sections were deparaffinised, rinsed and antigen retrieval was performed by incubating the slides for 20 min at 95°C in 0.01% citrate buffer. Sections were blocked in 5% NGS in 0.01% PBS/Tween 20 for 1 h. For detection of neutrophil and inflammatory monocyte infiltration into the heart, sections were then incubated overnight at 4°C with either Ly-6G^+^ (1:400 in 5% NGS and 0.01% PBS-T, BD Pharm, Scoresby, VIC, Australia) or Ly-6C^+^ primary antibody (1:400 in 5% NGS and 0.01% PBS-T, Bio-Rad, Hercules, CA, United States). Images were photographed and analyzed as described previously (Qin et al., 2017b).

Whole blood and plasma were collected from mice in cohort 2 to assess total and differential circulating white blood cells (WBC) numbers, as an assessment of systemic inflammation, as described previously (Qin et al., 2017a,b).

### Assessment of Cardiac Fibrosis and Apoptosis *in vivo*

Left ventricular tissues collected from mice in cohort 3 (following 40 min ischemia and 7-days reperfusion) were fixed in neutral-buffered formalin, embedded in paraffin (Alfred Pathology Service, Melbourne, VIC, Australia), sectioned at 4 μm and stained with picrosirius red (0.1%, Fluka, Bucks, Switzerland; pH2) for cardiac collagen deposition (Qin et al., 2017a,b). In addition, the level of apoptosis in LV was examined using CardiacTAC *In Situ* Apoptosis Detection kit (Trevigen, Gaithersburg, MD, United States). Images were photographed and analyzed as described previously (Qin et al., 2017a,b).

### Assessment of Cardiac Function by Echocardiography

M-mode echocardiography was performed in anesthetized mice (KXA: 80/8/0.96 mg/kg, i.p.) allocated to cohort 4, to obtain measures of LV function at 1 and 4 weeks post permanent ligation, utilizing a Philips iE33 ultrasound machine (North Ryde, NSW, Australia) with a 15 MHz linear transducer. Heart rate, left ventricular end-systolic dimension (LVESD), LV end-diastolic dimension (LVEDD), LV posterior wall thickness (LVPW) and fractional shortening (FS, %) were assessed from M-mode echocardiography as per the published guidelines for echocardiography in mice (Donner et al., 2018).

Left ventricular tissues were also collected from anesthetized mice in cohort 4 at study end point (4 weeks post MI). LV RNA was extracted from both the infarct and the non-infarcted AAR, and gene expression determined via qPCR as described previously (Qin et al., 2017a,b).

### Data Analysis

GraphPad Prism software (Version 7.00, La Jolla, CA, United States) was used to perform statistical analyses. Data are presented as expressed as mean ± SEM, unless otherwise stated. Statistical analyses utilized Student’s *t*-test or one-way ANOVA followed by Dunnett’s or Tukey’s *post hoc* test, as appropriate. Values of *P* < 0.05 were considered statistically significant. The Kaplan–Meier survival curve was analyzed by the log-rank (Mantel-Cox) test. Values of *P* < 0.05 were considered statistically significant for all analyses.

## Results

### Impact of Ac_2-26_ on Primary Cardiomyocytes and Cardiofibroblasts *in vitro*

**Figure 1:**
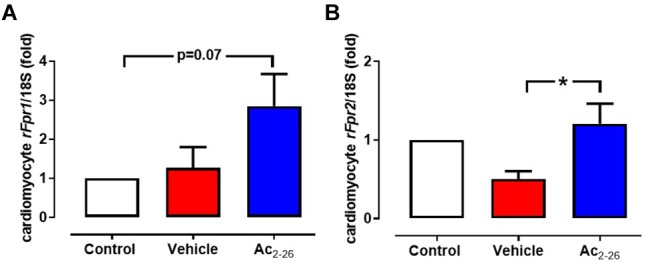
Ac_2-26_ increases cardiomyocyte expression of *rFprs.* Ac_2-26_ tends to increase expression of **(A)** *rFPR1* and **(B)** significantly increases *rFPR2* expression. ^∗^*P* < 0.05 versus paired vehicle-treated mice, one-way repeated measures ANOVA with Dunnett’s *post hoc* test. n (number of different cardiomyocyte preparations) = 5. Data were presented as mean ± SEM.

After 48 h incubation of cardiomyocytes with Ac_2-26,_ the expression of *rFpr1, rFpr2, and rFPR3* were assessed. Low levels of constitutive expression of both *rFpr1* (Ct∼33) and *rFpr2* (Ct∼34) were detected in untreated cardiomyocytes. Ac_2-26_ tended to increase *rFpr1* (Figure 1A, *p* = 0.05, overall on one-way ANOVA with Dunnett’s *post hoc* test) and significantly increased *rFpr2* expression (Figure 1B, *p* = 0.04, one-way ANOVA with Dunnett’s *post hoc* test) compared to vehicle-treated cardiomyocytes. Expression of *rFpr3* was only detectable in 2 out of 5 cardiomyocytes preparations, the average of *rFpr3* expression was 0.3- and 3.0-fold untreated cardiomycytes after vehicle or Ac_2-26_ in these two preparations.

**Figure 2:**
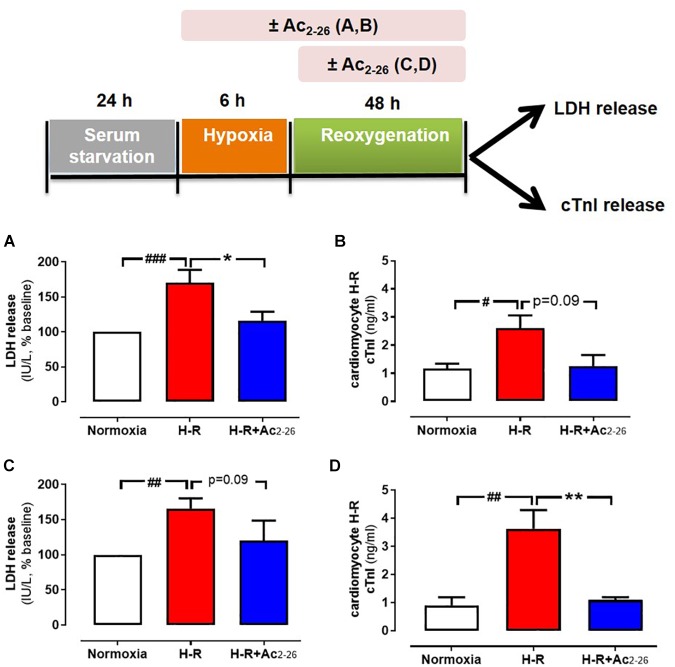
Protective actions of Ac_2-26_ against cardiomyocyte injury responses *in vitro.* Ac_2-26_ (1 μM) prevents neonatal rat cardiomyocyte hypoxia-reoxygenation (H-R) injury *in vitro* (assessed by measuring LDH and cTnI release), whether present for the full duration of H-R (**A,B**, *n* = 7 cardiomyocyte preparation) or only post H-R (**C,D**, *n* = 5 cardiomyocyte preparation). ^#^*P* < 0.05, ^##^*P* < 0.01, ^###^*P* < 0.001 versus normoxia, ^∗^*P* < 0.05 ^∗∗^*P* < 0.01 versus vehicle-treated mice from the same cardiomyocyte preparation. One-way ANOVA with Dunnett’s *post hoc* test. n (number of different cardiomyocyte preparations) = 5. Data were presented as mean ± SEM.

**Figure 3:**
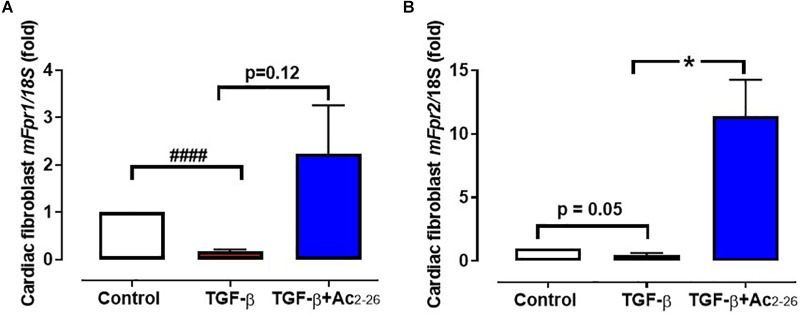
Effect of Ac_2-26_ on TGF-β stimulation of cardiofibroblasts *in vitro.* TGF-β (10 μM) downregulates **(A)** *mFpr1* and **(B)** *mFpf2* expression, which is elevated by Ac_2-26_ (10 μM) within 48 h. ^####^*P* < 0.001 versus control, ^∗^*P* < 0.05 versus TGF-β-treated cells from the same cardiofibroblast preparation. One-way ANOVA with Dunnett’s *post-hoc* test. n (number of different cardiofibroblasts preparations) = 7. Data were presented as mean ± SEM.

The direct effects of Ac_2-26_ in primary cardiomyocytes and cardiofibroblasts were determined *in vitro*. Hypoxia-reoxygenation (H-R) significantly increased LDH release (Figure 2A, *p* = 0.01 overall on one-way ANOVA) from cardiomyocytes by approximately 70%, while cTnI (a more sensitive measure of cardiomyocyte death) (Figure 2B, *p* = 0.01 overall on one-way ANOVA, and on Dunnett’s *post hoc* test) was almost tripled, compared to control cardiomyocytes. Ac_2-26_ (1 μM) prevented cardiomyocyte LDH and cTnI release whether present for the full duration of H-R (Figure 2A,B), or when only added at the start of reoxygenation (Figure 2C, *p* = 0.005 overall on one-way ANOVA and on Dunnett’s *post hoc* test; Figure 2D, *p* = 0.0009 overall on one-way ANOVA). These results confirm that Ac_2-26_ elicits direct protective actions on cardiomyocytes subjected to H-R, and that these are comparable whether treatment is commenced concomitantly with the insult, or at the onset of post-insult recovery. We also sought the impact of Ac_2-26_ on cardiac fibroblast FPR gene expression *in vitro*, and observed that Ac_2-26_ tends to upregulate both *mFpr1* and *mFpr2* (Figure 3A,B); *mFpr2* was significantly upregulated > 10-fold (Figure 3B, *p* = 0.04 overall on one-way ANOVA and on Dunnett’s *post hoc* test), with a moderate but non-significant tendency for increased *mFpr1* (Figure 3A, *p* = 0.09 overall on one-way ANOVA, and on Dunnett’s *post hoc* test).

### Ac_2-26_ Significantly Reduces Myocardial Necrosis *in vivo*

To assess the impact of Ac_2-26_ in an animal model of myocardial I-R *in vivo*, C57BL/6 mice underwent reversible ligation of the LAD coronary artery followed by reperfusion in the absence or presence of treatment for 24 h (cohort 1), 48 h (cohort 2), or 7-days (cohort 3).

**Figure 4:**
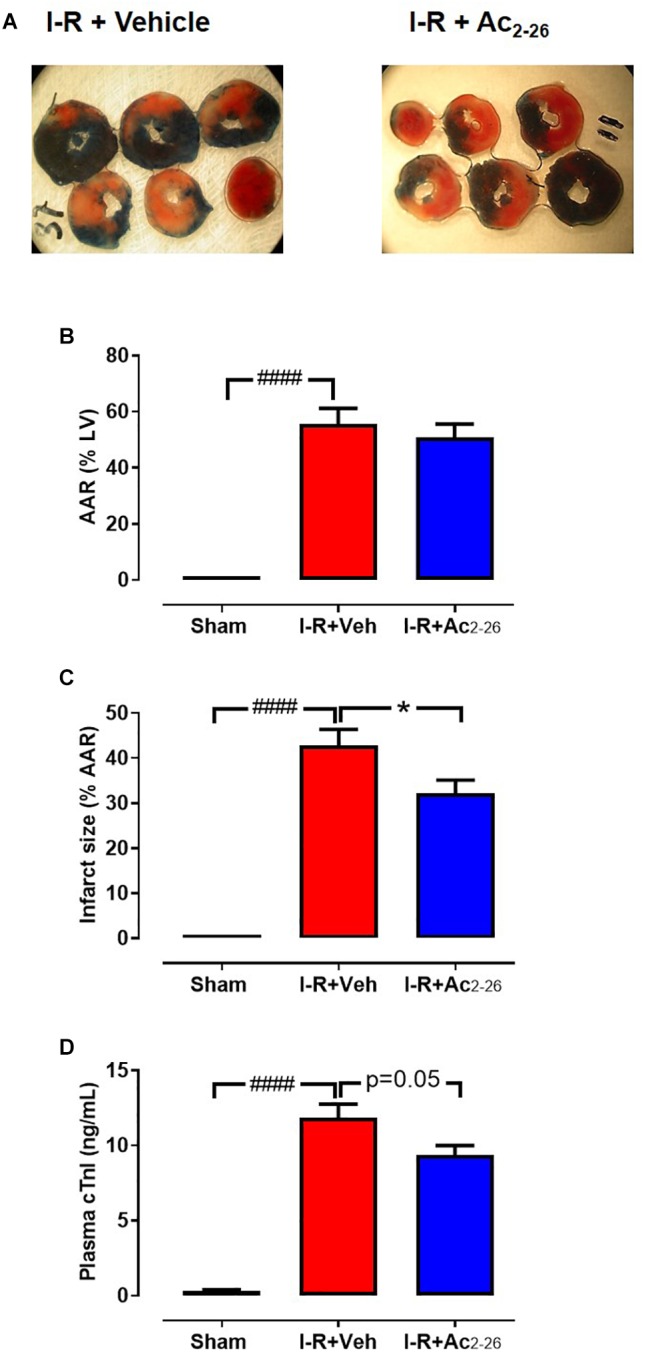
Ac_2-26_ reduces cardiac necrosis 24 h post I-R injury *in vivo*. **(A)** LV transverse slices from representative mice subjected to 40 min ischemia followed by 24 h reperfusion following either vehicle or Ac_2-26_ (1 mg/kg i.p. administered at time of reperfusion). Three different zones are visible after staining with Evans blue and 2,3,5-triphenyltetrazolium chloride. Areas stained dark blue, white and red represented non-risk, infarcted and ischemic but non-infarcted zones, respectively. The risk zone includes red and white areas. **(B)** Area-at-risk (AAR) in mice subjected to myocardial I-R, **(C)** Infarct size, and **(D)** Plasma levels of cardiac troponin (cTnI). ^####^*P* < 0.001 versus sham, ^∗^*P* < 0.05 versus vehicle-treated mice. One-way ANOVA with Tukey *post hoc* test. Data were presented as mean ± SEM; with number of mice per group. *n* = 3 (sham), *n* = 5 (I-R + vehicle), and *n* = 12 (I-R + Ac_2-26_).

The effect of Ac_2-26_ (1 mg/kg, i.v.) on cardiac necrosis was assessed in mice subjected to 40 min ischemia-24 h reperfusion (cohort 1). There were no differences between I-R groups in the identified size of the LV AAR (∼60%) after 24 h reperfusion, as measured by Evans blue staining (Figure 4A,B, *p* < 0.0001 overall on one-way ANOVA). Of the AAR in vehicle-treated I-R mice, 30–40% was infarcted (Figure 4C, *p* < 0.0001 overall on one-way ANOVA), and plasma levels of cTnI were markedly elevated (Figure 4D, *p* < 0.0001 overall on one-way ANOVA). Administration of Ac_2-26_ peptide immediately before LAD reperfusion significantly attenuated infarct size by ∼25%, and tended to lower cTnI levels, relative to vehicle-treated I-R injured mice (*p* = 0.05, one-way ANOVA with Dunnett’s *post hoc* test, Figure 4D).

### Ac_2-26_ Attenuates Cardiac and Systemic Inflammation *in vivo*

**Figure 5:**
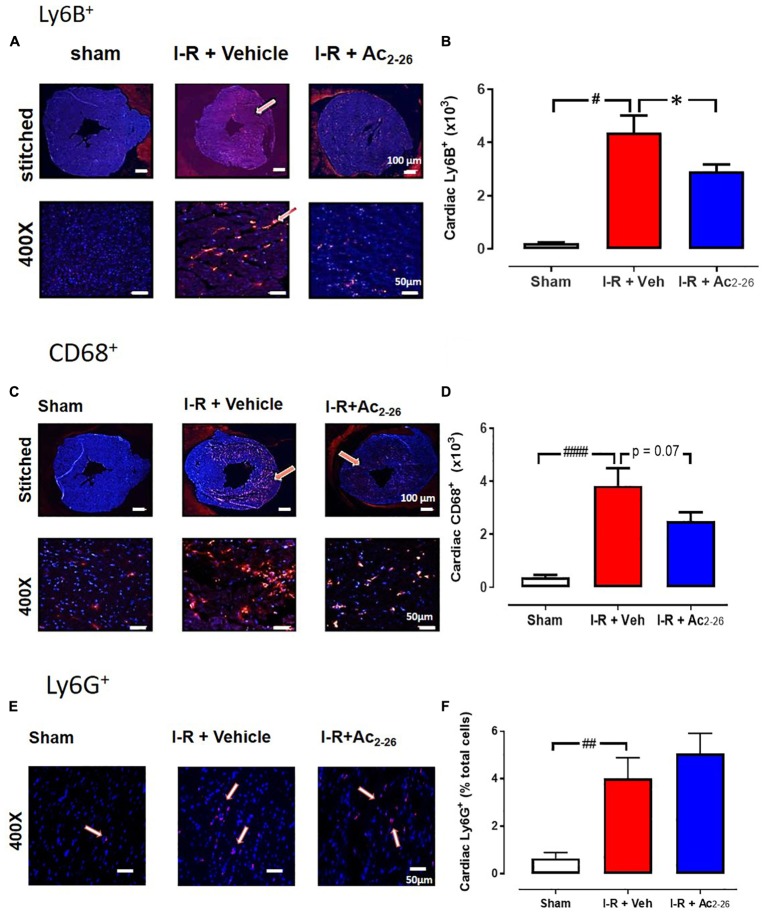
Ac_2-26_ reduces cardiac inflammation 48 h post I-R injury *in vivo*. Representative immunofluorescent images of **(A)** LV inflammatory cell content (determined using anti-Ly-6B.2 antibody, **(C)** LV macrophage content (determined using anti-CD68 antibody, **E**) LV neutrophil content (determined using anti-Ly6G^+^ antibody) from sham, vehicle- and Ac_2-26_-(1 mg/kg/day, i.v.)-treated mice, 48 h post I-R (X400 magnification, scale-bar in top panels = 100 μm; in bottom panels = 50 μm). Pooled data for **(B)** LV Ly6B.2 positive, **(D)** C68^+^ positive, and **(F)** Ly6G^+^ positive immunofluorescence. Higher magnification images reveals overlay of dark blue (DAPI; detecting nuclei) and red (inflammatory cells) indicating positive staining (scale-bar in top panels = 100 μm; in bottom panels = 50 μm). ^#^*P* < 0.05, ^##^*P* < 0.01, ^####^*P* < 0.0001 versus sham, ^∗^*P* < 0.05 versus vehicle-treated mice. One-way ANOVA with Tukey’s *post hoc* test. Data were presented as mean ± SEM, with number of mice per group. *n* = 6 (sham), *n* = 7 (I-R + vehicle), and *n* = 8 (I-R + Ac_2-26_).

**Figure 6:**
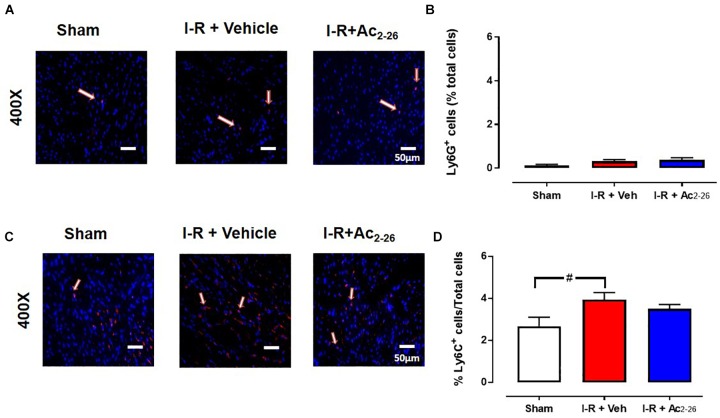
Ac_2-26_ has no impact on myocardial neutrophil content 7 days post I-R injury *in vivo*. Representative immunofluorescent images of **(A)** LV neutrophil content (determined using anti-Ly6G^+^ antibody, and **C**) inflammatory monocytes (determined using anti-Ly6C^+^ antibody). Pooled data for both are shown in **B**) LV LyG^+^ and **(D)** LV Ly6C^+^ immunofluorescence. Images reveals overlay of dark blue (DAPI; detecting nuclei) and red (inflammatory cells) indicating positive staining (scale-bar = 50 μm). ^#^*P* < 0.05 versus sham. One-way ANOVA with Tukey’s *post hoc* test. Data were presented as mean ± SEM, with number of mice per group. *n* = 7 (sham), *n* = 4 (I-R + vehicle), and *n* = 6 (I-R + Ac_2-26_).

The extent of I-R-induced cardiac injury and systemic inflammation was assessed after 48 h and 7 days reperfusion *in vivo*, as well as the impact of Ac_2-26_ on these changes (cohort 2 and cohort 3). As shown via immunofluorescent detection of LV inflammatory cells (anti-Ly-6B.2), I-R injury elicited a significant increase in inflammatory cells signals per section, in both vehicle- and Ac_2-26_-treated I-R mice, compared to sham (Figure 5A,B, *p* < 0.0001 overall on one-way ANOVA). Administration of Ac_2-26_ reduced cardiac neutrophil numbers by approximately 35% compared to vehicle-treated I-R (Figure 5A). Macrophage numbers in LV sections, detected using anti-CD68, were also increased in mice subjected to I-R (Figure 5C,D, *p* < 0.0001 on one-way ANOVA); Ac_2-26_ tended to blunt the increase in LV macrophage density (*p* = 0.10, one-way ANOVA with Dunnett’s *post hoc* test, Figure 5D). Consistent with the Ly6B results, accumulation of Ly6G (indicative of neutrophils) was also significantly increased post ischemic insult (Figure 5E,F). Ac_2-26_ failed to exert impact on LV neutrophil content at this 48 h timepoint. Interestingly, levels of Ly6G-positive cells appear to have returned to baseline levels by 7 days post I-R (Figure 6A,B). Levels of Ly6C-positive cells (inflammatory monocytes) is significantly elevated 7 days post-ischemic insult (Figure 6C,D). Administration of Ac_2-26,_ however, exerted no impact on the total LV content of Ly6G- and Ly6C-positive cells 7 days post I-R (Figure 6).

**Figure 7:**
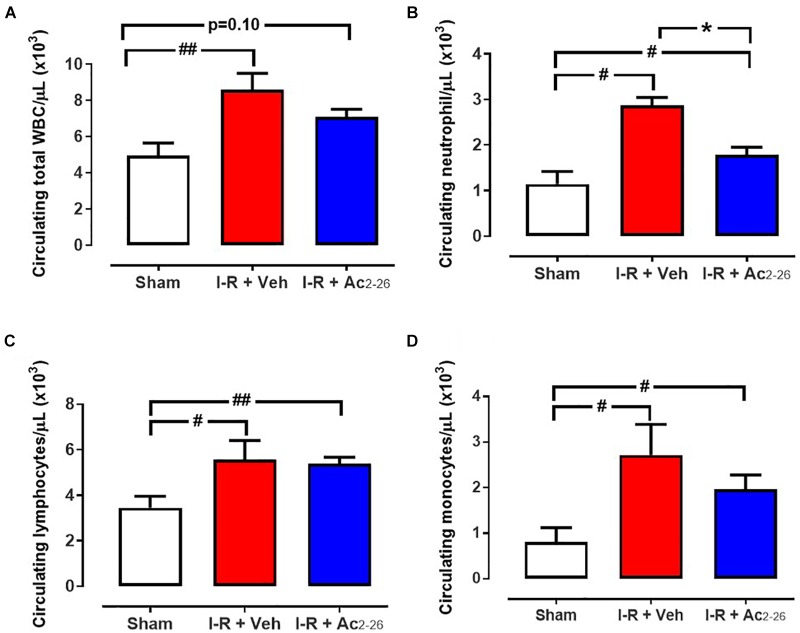
Ac_2-26_ reduces systemic inflammation 48 h post I-R injury *in vivo*. Circulating levels of **(A)** total white blood cells (WBC), **(B)** neutrophils, **(C)** lymphocytes and **(D)** monocytes in blood collected from sham, vehicle- and Ac_2-26_-(1 mg/kg/day, i.v.)-treated mice, 48 h post I-R. ^#^*P* < 0.05, ^##^*P* < 0.01 versus sham, ^∗^*P* < 0.05 versus vehicle-treated mice. One-way ANOVA with Dunnett’s *post hoc* test. Data were presented as mean ± SEM, with number of mice per group. *n* = 6 (sham), *n* = 7 (I-R + vehicle), and *n* = 8 (I-R + Ac_2-26_).

Total and differential WBC quantification were performed, to investigate the effects of Ac_2-26_ on systemic inflammation (Figure 7). Circulating total WBCs (*p* = 0.009 overall on one-way ANOVA), lymphocytes (*p* = 0.04 overall on one-way ANOVA), neutrophils (*p* = 0.0005 overall on one-way ANOVA) and monocytes (*p* = 0.03 overall on one-way ANOVA) were all significantly increased in I-R vehicle-treated mice compared with sham (Figure 7). Ac_2-26_ significantly blunted the 3-fold increase in levels of circulating neutrophils (Figure 7B), without reducing the I-R induced increases in circulating lymphocytes and monocytes (Figure 7C,D).

### Ac_2-26_ Limits Cardiac Remodeling *in vivo*

**Figure 8:**
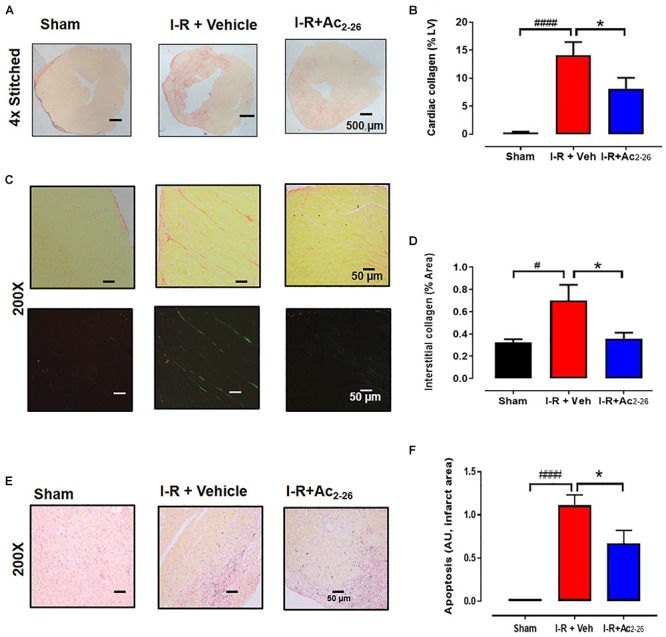
Ac_2-26_ reduces cardiac fibrosis at 7 days post I-R injury *in vivo*. **(A)** Representative picrosirius red-stained LV cross-sections from sham, vehicle- and Ac_2-26_-(1mg/kg/day, i.v.)-treated mice, 7-days post I-R (collagen appears red); **(B)** quantification of cardiac fibrosis. **(C)** Representative picrosirius red-stained remote area (bright field and polarized light) from sham, vehicle- and Ac_2-26_-(1mg/kg/day, i.v.)-treated mice, 7-days post I-R, **(D)** quantification of interstitial fibrosis under polarized light, magnification x200. **(E)** Representative CardioTAC-stained LV cross-sections from sham, vehicle- and Ac_2-26_-(1 mg/kg/day, i.v.)-treated mice, 7-days post I-R (magnification x200). **(F)** Quantification of dead:viable cells (expressed as fold change versus sham; magnification x200. ^#^*P* < 0.05, ^####^*P* < 0.0001 versus sham, ^∗^*P* < 0.05 versus vehicle-treated I-R mice. One-way ANOVA with Tukey’s *post hoc* test. Data were presented as mean ± SEM, with number of mice per group. *n* = 4 (sham), *n* = 6 (I-R + vehicle), and *n* = 6 (I-R + Ac_2-26_).

Next, the impact of Ac_2-26_ on I-R-induced cardiac fibrosis (by picrosirius red, Figure 8A–D) and cardiac apoptotic cell death (by CardioTACS, Figure 8E,F) after 40 min ischemia with 7-days reperfusion was determined (cohort 3). Myocardial I-R significantly increased collagen deposition (Figure 8A, *p* < 0.001 overall on one-way ANOVA). Our data shows that cardiac collagen (% LV area) is significantly increased in the LV of vehicle-treated I-R mice compared to sham; this was attenuated by Ac_2-26_ treatment (Figure 8B). Quantitative analysis revealed that the interstitial staining (% area) in the remote zone is more than doubled in vehicle-treated I-R mice compared to sham; this increase was completely abolished by Ac_2-26_ treatment (Figure 8C,D, *p* = 0.04 overall on one-way ANOVA). Apoptosis was not evident in cardiac sections from sham mice. Myocardial I-R significantly increased LV cardiac cell death, which was significantly attenuated by Ac_2-26_ treatment (Figure 8C,D, *p* < 0.0001 overall on one-way ANOVA).

### Impact of Ac_2-26_ on Organ Weights After I-R Injury *in vivo*

Body and organ weights measured after 48 h (cohort 2) and 7-day (cohort 3) reperfusion are shown in Table 2. Following 48 h post-ischemic reperfusion, no differences in body weight (BW), atria or lung weights were observed, but heart weight (HW) was significantly increased in both vehicle and Ac_2-26_-treated hearts compared to sham (cardiac weights were normalized to BW). The increase in LV:BW ratio after 7-day reperfusion with vehicle-treated I-R compared with sham mice was not prevented by Ac_2-26_ treatment (Table 2).

**Table 2:** Impact of I-R injury after either vehicle- or Ac_2-26_ treatment, on body and organ weights at endpoint, either 48 h or 7-days following I-R.

		Sham	I-R + vehicle	I-R + Ac_2-26_
**48 h myocardial I-R**				
*n*		**6**	**7**	**8**
Body Weight (BW, g)		26.9 ± 1.3	29.0 ± 0.4	28.2 ± 1.0
Organ weight/BW (mg/g)	Heart	4.2 ± 0.1	4.8 ± 0.2^#^	5.0 ± 0.2^#^
	Atria	0.3 ± 0.0	0.4 ± 0.0	0.3 ± 0.0
	LV	3.1 ± 0.1	3.6 ± 0.1	3.8 ± 0.2
	RV	0.8 ± 0.1	0.8 ± 0.1	0.8 ± 0.0
	lung	5.2 ± 0.2	5.3 ± 0.1	5.0 ± 0.3
**7-days myocardial I-R**				
*n*		**4**	**6**	**6**
Body Weight (BW, g)		26.3 ± 0.7	25.5 ± 0.6	25.6 ± 1.0
Organ weight/BW (mg/g)	Heart	4.4 ± 0.2	5.2 ± 0.2^#^	5.0 ± 0.2^#^
	Atria	0.2 ± 0.0	0.4 ± 0.00^#^	0.3 ± 0.0
	LV	3.4 ± 0.1	3.9 ± 0.2^#^	4.0 ± 0.2^#^
	RV	0.8 ± 0.1	0.9 ± 0.1	0.7 ± 0.1
	Lung	5.8 ± 0.3	5.6 ± 0.16	5.7 ± 0.2

^#^*P < 0.05 vs. sham. One-way ANOVA with Dunnett’s *post-hoc* test. Data were presented as mean ± SEM, *n* indicates number of mice at endpoint*.

### Impact of Ac_2-26_ on MI-Induced Cardiac Dysfunction *in vivo*

**Figure 9:**
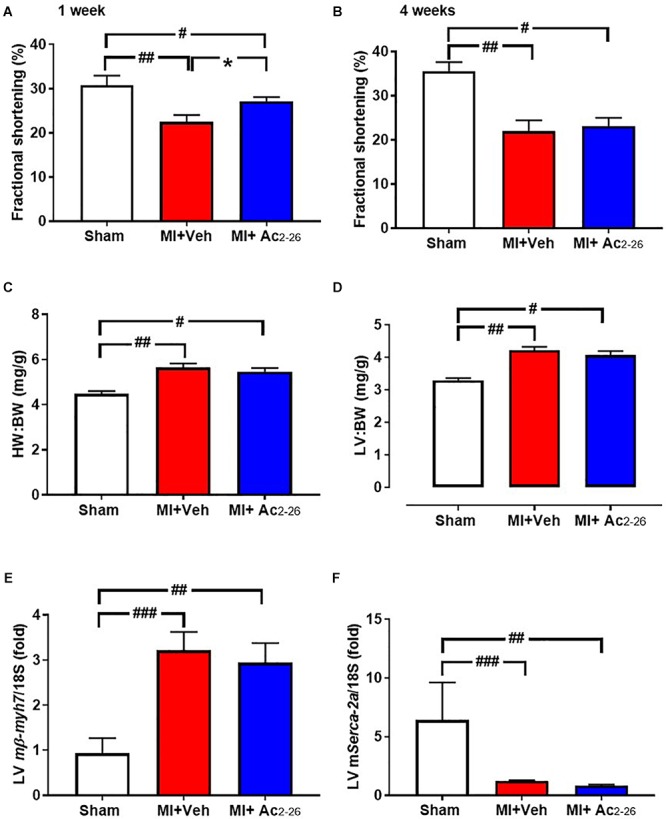
Impact of Ac_2-26_ on cardiac response to MI over the long-term. Cardiac dysfunction was assessed after 4 weeks of permanent LAD occlusion. MI significantly impaired fractional shortening **(A)** 1 week and **(B)** 4 weeks post LAD ligation. **(C)** HW:BW, **(D)** LV:BW and **(E)** LV *mmyh7* were increased and **(F)** LV m*Serca-2a* was decreased in response to MI. Administration of Ac_2-26_ significantly improved cardiac function 1 week post LAD ligation **(A)**, but not other changes in parameters 4 weeks post MI **(B–F)**. ^#^*P* < 0.05, ^##^*P* < 0.01, ^###^*P* < 0.001 vs. sham and ^∗^*P* < 0.05 vs. vehicle-treated MI mice. One-way ANOVA with Tukey’s *post-hoc* test. Data were presented as mean ± SEM, with number of mice per group. *n* = 6 (sham), *n* = 10 (I-R + vehicle), and *n* = 12 (I-R + Ac_2-26_).

The effects of Ac_2-26_ were assessed on severe cardiac injury was induced by 4 weeks permanent LAD occlusion (cohort 4). All mice in this cohort were included in survival analysis (see Supplementary Figures S1, S2). There was no statistical difference in survival between vehicle and Ac_2-26_ treated mice after MI (Supplementary Figure S2). End-point body and organ weights after sham surgery or 4 weeks post-MI, are shown in Table 3. Surgical MI or treatment with Ac_2-26_ did not affect BW or normalized RV or lung weight. HW, LV and atria weights were significantly increased to similar levels in both vehicle and Ac_2-26_-treated hearts (Figure 9 and Table 3).

**Table 3:** Impact of MI *in vivo* in the absence and presence of Ac_2-26_ administration, on endpoint body and organ weights 4 weeks following permanent LAD occlusion, as well as echocardiographic parameters of LV function in anesthetized mice at 1 and 4 weeks post MI.

		Sham	MI	MI+ Ac_2-26_
*n*		**6**	**10**	**12**
Body Weight (BW, g)		28.5 ± 0.7	28.3 ± 0.5	29.0 ± 0.5
Organ weight/BW (mg/g)	Atria	0.3 ± 0.0	0.5 ± 0.0^#^	0.5 ± 0.0^#^
	RV	0.8 ± 0.1	0.9 ± 0.1	0.9 ± 0.0
	Lung	5.9 ± 0.2	6.2 ± 0.2^#^	6.4 ± 0.2^#^
**Echocardiographic analysis (1 week post MI)**				
		**Sham**	**MI**	**MI+ Ac_2-26_**
HR (beats per min)		518 ± 32	572 ± 24	551 ± 12
LVEDD (mm)		4.27 ± 0.23	4.65 ± 0.16	4.55 ± 0.07
LVPW (mm)		0.64 ± 0.02	0.67 ± 0.02	0.71 ± 0.02
LVESD (mm)		2.97 ± 0.24	3.62 ± 0.17^#^	3.31 ± 0.07
**Echocardiographic analysis (4 weeks post MI)**				
HR (beats per min)		608 ± 7	582 ± 20	600 ± 18
LVEDD (mm)		4.03 ± 0.08	4.92 ± 0.19^##^	4.73 ± 0.14^#^
LVPW (mm)		0.68 ± 0.02	0.79 ± 0.02	0.77 ± 0.02
LVESD (mm)		2.59 ± 0.10	3.86 ± 0.22^###^	3.65 ± 0.16^##^

*LVEDD, LV end-diastolic dimension; LVESD, LV end-systolic dimension; LVPW, LV posterior wall thickness;^#^*P* < 0.05, ^##^*P* < 0.01, ^###^*P* < 0.001 vs. sham. One-way ANOVA with Dunnett’s *post hoc* test. Data were presented as mean ± SEM, *n* indicates number of mice at endpoint*.

Echocardiographic analysis of cardiac dimensions and LV function was performed in anesthetized mice both at 1 week post MI and at study end point (Table 3 and Figure 9 LV chamber dimensions measured as LVESD and LVEDD thickness were increased by MI 4 wks post MI (Table 3). Although there was no change in heart rate, %FS was reduced at both 1 and 4 weeks post MI (Figure 9A,B). This was partially protected with 1 week, but not 4 weeks, of Ac_2-26_ treatment. The significant MI-induced increase in hypertrophic *mmyh7*-gene expression, and reduction in LV sarco/endoplasmic reticulum Ca^2+^-ATPase (*mSerca-2a* gene expression), both of which play an important role in cardiac contractile function, were not prevented by Ac_2-26_ treatment (Figure 9E *p* = 0.01 overall on one-way ANOVA; Figure 9F, *p* = 0.005 overall on one-way ANOVA). In addition, the MI-induced increase in pro-fibrotic *mCtgf* gene expression in the non-infarct area at the 28-day timepoint was no longer evident in mice administered Ac_2-26_ (Supplementary Table S2). Ac_2-26_ also significantly reduced transforming growth factor-β (*mTgf*-β expression; Supplementary Table S2). Further, the significantly increased gene expression of the pro-inflammatory macrophage marker *mS100a9* at this timepoint (*p* < 0.05) was no longer evident in mice treated with Ac_2-26_ for 4 weeks post MI (Supplementary Table S2).

## Discussion

**Figure 10:**
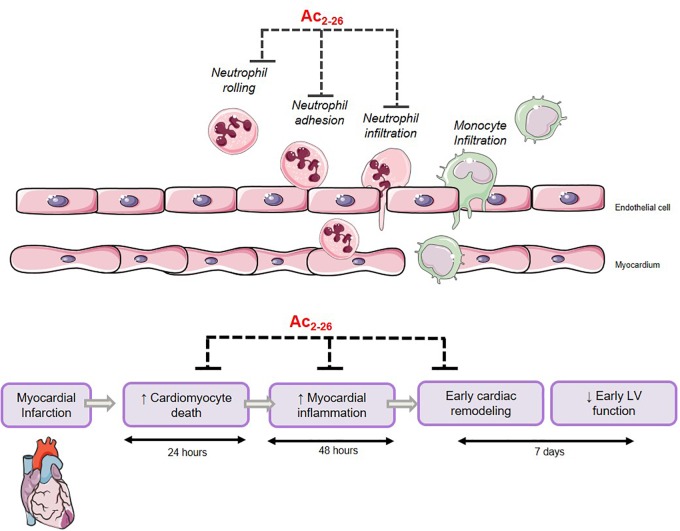
Proposed mechanism of Ac_2-26_-induced cardioprotection. Ac_2-26_ reduces cardiomyocyte death, myocardial inflammation, and limits early cardiac remodeling against myocardial ischemic insult. Schema created using a modification to an image provided by Servier Medical Art by Servier (https://smart.servier.com/image-set-download/), licensed under a Creative Commons Attribution 3.0 Unported Licence (https://creativecommons.org/licences/by/3.0/).

The major finding of the present study revealed that the N-terminal peptide of ANX-A1, Ac_2-26_, not only reduces cardiomyocyte death *in vitro*, but also reduces cardiac necrosis, inflammation, cardiac fibrosis and apoptosis, in a preclinical mouse model of myocardial I-R injury as summarized in Figure 10. Further, Ac_2-26_ delays the onset of cardiac dysfunction after MI in mice *in vivo*. These early cardioprotective properties of Ac_2-26_ in the first few days after myocardial ischemic insults in particular may reveal clinical insights into protective add-on approaches for management of MI.

We have previously demonstrated that FPRs are functionally expressed in the whole heart and in isolated cardiomyocyte preparations (Qin et al., 2013). Given that our prior *in vitro* experiments lacked the concomitant presence of immune cells, it is thus likely that the cardioprotective action of Ac_2-26_ *in vivo* could be mediated via both FPR1- and FPR2-dependent mechanisms *in vivo* (Qin et al., 2015). We have previously shown that administration of Ac_2-26_ at the time of reperfusion *in vitro* preserves LV contractile function in isolated buffer-perfused rodents hearts subjected to I-R, via an FPR1-dependent mechanism (Qin et al., 2013). In contrast, the anti-inflammatory effects of Ac_2-26_ are considered predominantly mediated via FPR2 (Gavins et al., 2003a). Here, we investigated the impact of Ac_2-26_ on gene expression of both FPR1 and FPR2 subtypes in cardiomyocytes, and observed that Ac_2-26_ significantly increased FPR2 gene expression over 48 h. These data are consistent with an acute Ac_2-26_-mediated activation of cardiac FPRs to preserve LV function (within minutes, especially FPR1), whilst stabilizing cardiac FPR mRNA expression within days following its administration, especially given the reported short (90 min) half-life of FPR mRNA in unstimulated cells (Mandal et al., 2005; Waechter et al., 2012).

A number of studies have previously investigated the cardioprotective actions of Ac_2-26_ and full-length ANX-A1 protein *in vivo* (La et al., 2001a; Gavins et al., 2005; D’Amico et al., 2000; Dalli et al., 2012), at least over the short-term. The duration of reperfusion following ischemia employed in these earlier I-R studies *in vivo* has ranged from 45 min to 2 h (La et al., 2001a; Gavins et al., 2003a, 2005). A small number of studies have shown that Ac_2-26_ reduces infarct size and leukocyte recruitment when administrated at reperfusion (D’Amico et al., 2000; La et al., 2001b; Gavins et al., 2005). However, the cardioprotective effect of Ac_2-26_ against myocardial I-R injury at later timepoints (beyond the first few hours) has not been previously determined. The current study demonstrated for the first time that Ac_2-26_ reduces myocardial infarct size, and levels of the circulating cardiomyocyte injury marker, cTnl, 24 h post I-R, indicating that Ac_2-26_ likely preserves cardiomyocyte viability *in vivo*. This suggests that Ac_2-26_ might offer potential protection of cardiomyocyte viability in clinical settings.

Inflammatory cell infiltration into the myocardium is observed early, in the first few hours of reperfusion following ischemia (Dreyer et al., 1991; Hawkins et al., 1996; La et al., 2001b; Gavins et al., 2005). Infiltrating neutrophils play a central role in damage of MI. They are the first cells to be summoned to the insult region, resulting in exaggerated levels of both reactive oxygen species (ROS) and pro-inflammatory responses, leading to microvascular injury, cardiomyocyte death, extracellular matrix degradation and adverse cardiac remodeling (Gao et al., 2012). Neutrophil activation in MI promotes expression of adhesion molecules, leading to adherence of neutrophils to the endothelium, followed by transmigration, and direct interaction with cardiomyocytes (Ansari et al., 2018). Several studies have demonstrated the anti-neutrophil potential effects of ANX-A1 and Ac_2-26._ Administration of Ac_2-26_ was previously shown to reduce neutrophil infiltration into cardiac tissue after 1 h reperfusion in a mouse model of I-R (Gavins et al., 2006). The resultant increased cardiac accumulation of neutrophils and macrophages likely contributes to myocardial damage following the insult, consistent with our observations in the present study, 48 h after I-R. Our study now demonstrates that Ac_2-26_-induced significant reduction of local myocardial inflammatory cell infiltration, especially neutrophils, is still evident at a much later timepoint than previously observed, consistent with the early inhibition of neutrophil infiltration into mouse myocardial injury after the insult (Gavins et al., 2003a).

In the present study, a significant increase in circulating total and differential WBC following 48 h reperfusion was observed in response to I-R in mice, predominately attributed to increased circulating lymphocytes and neutrophils. Ac_2-26_ administration significantly reduced circulating WBC and neutrophils, suggesting this peptide may reduce systemic inflammation in response to an ischemic insult. An ischemic insult stimulates the expansion and mobilization of haematopoietic stem progenitor cells (HSPCs), to increase supply of myeloid cells to the injured myocardium (Dutta et al., 2012); endogenous ANX-A1 plays an important role in this progress (Qin et al., 2017a). It is thus possible that Ac_2-26_ may limit HSPCs expansion and mobilization, however, this was beyond the scope of the current study.

In this study, we have measured the levels of leukocytes (Ly6B^+^), macrophage (CD68^+^) neutrophil (Ly6G^+^) and inflammatory monocytes (Ly6C^+^) 48 h and 7 days post ischemic insult (Lee et al., 2013). In our hands, levels of infiltrating inflammatory cells peaks at 48 h, whilst levels of inflammatory monocytes/macrophages remain elevated 7 days after the insult. Ac_2-26_ reduced the total number of leucocytes, especially macrophages, but not the total number of neutrophils infiltrating the heart. However, the function of neutrophils could be affected by Annexin A1 and Ac_2-26,_ as has been demonstrated in human neutrophils previously (Dalli et al., 2012); this was not, however, sought here.

Cardiac fibrosis is a key hallmark of the progression to HF following ischemic insults (Gao et al., 2012). This is attributed to necrotic and apoptotic cardiomyocytes being replaced by extracellular matrix proteins following the injury. In the present study, a significant increase in LV collagen deposition was clearly evident 7-days post I-R. Excitingly, our analysis revealed that Ac_2-26_ blunted this I-R-induced LV collagen deposition, suggesting that Ac_2-26_ has the potential to elicit relatively long-term cardioprotective effects, particularly at the level of adverse remodeling. Cardiomyocyte apoptosis was also significantly elevated in mice 7-days post I-R, consistent with previous reports (Qin et al., 2017b). This was significantly reduced by Ac_2-26_, suggesting that Ac_2-26_ may be able to enhance the survival of cardiomyocytes.

Left ventricular contractile impairment is a causal contribution of progression to HF following ischemic injury (Gao et al., 2012). In the present study, Ac_2-26_ delayed cardiac dysfunction in response to permanent LAD occlusion, as observed by protection of FS 1 week after MI, whilst this was no longer apparent after 4 weeks. This is consistent with our previous report that the permanent ligation-induced impairment in cardiac function was only significantly exaggerated in ANX-A1 deficient mice at 1 week, but not 4 weeks, post MI (Qin et al., 2017a). It is also possible that the full length ANX-A1 protein (or other, more stable, FPR agonists) is required to rescue the cardiac function over a longer-term, as suggested by previous reports of cardioprotection with the small-molecule FPR agonist Cmpd17b (Qin et al., 2017b) and the cleavage resistant peptide, CR-Ac_2-50_ (Dalli et al., 2013).

### Limitations of the Study

In this study, we demonstrated that Ac_2-26_ attenuates cardiac necrosis, inflammation, and early remodeling against myocardial insults *in vitro and in vivo*. Whilst Ac_2-26_ delayed cardiac dysfunction by 1 week, we did not obtain additional echocardiographs between the 1 and 4 week timepoints, thus the maximum amount of time that cardiac dysfunction was delayed by the intervention could not be determined. In addition, higher doses, or more metabolically stable forms, of Ac_2-26,_, which might provide additional protection of LV function over the longer-term (Dalli et al., 2013) were not investigated here. It is also important to note that determination of gene expression in ischemic myocardium at the end of 4 weeks post MI, such as in this study, may be limited by the tissue being too necrotic and/or fibrotic for treatment modalities to penetrate. However, examination of gene expression at earlier timepoints, or within the at-risk but non-infarcted myocardium, have provided additional insights into the cardioprotection mechanisms of Ac_2-26_. Further, changes observed at the level of gene expression are not always accompanied by parallel changes in protein levels. The level of FPR expression in the heart is widely accepted to be relatively low compared to inflammatory cells and other tissues, e.g., kidney (Qin et al., 2015). Whilst only levels of FPR gene expression (and not protein) were reported in the present study, this was precluded by the less sensitive nature of conventional protein detection methods such as immunohistochemistry or Western blot, compared to qPCR measurement of gene expression, regardless of the target assessed. Lastly, we suggest that future studies explore more detailed mechanistic interrogation, to further elucidate the mechanism which ANX-A1 and its mimetics might promote active resolution in the injured myocardium. In addition, whether delayed intervention of Ac_2-26_ (e.g., 1–4 h post MI), in keeping with the timing of percutaneous intervention in the clinic, could enhance its translational therapeutic potential.

## Conclusion

This study suggests that the ANX-A1 system may represent a novel therapeutic target for ischemic heart disease, given its ability to abrogate cardiomyocyte necrosis, inflammation and remodeling in a relevant preclinical model *in vivo* (illustrated in Figure 10). We have also provided new insights into the opportunities offered by ANX-A1-based approaches to limit early cardiac contractile dysfunction after an ischemic insult *in vivo*. This delay in progression of LV systolic dysfunction is a likely consequence of the favorable pro-resolving effects of Ac_2-26_. Development of ANX-A1 mimetics that are resistant to degradation are particularly attractive for future translational studies, particularly for treating MI in the early hours after an ischemic event (while the injury is still evolving), alone or concurrent with standard care, to reduce progression to HF and death in affected patients.

## Author Contributions

CQ, XG, and RR conceived and designed the study. CQ, SR, NC, MD, JW, AA, DH, RL, HK, X-JD, and XG performed the experiments. CQ, NC, MD, MT, RL, JW, DD, and RR analyzed the data. CQ, XG, X-JD, XG, and RR interpreted the results. CQ and JW prepared the figures. CQ and RR drafted the manuscript. CQ, JB, YY, DD, HK, ML, AM, X-JD, XG, and RR edited and revised the manuscript.

## Conflict of Interest Statement

The authors declare that the research was conducted in the absence of any commercial or financial relationships that could be construed as a potential conflict of interest.
