# Supplementary material for: Cardioprotective Actions of the Annexin-A1 N-Terminal Peptide, Ac2-26, Against Myocardial Infarction
Source: Front Pharmacol. 2019 Apr 3;10:269. doi: 10.3389/fphar.2019.00269 (PMC6457169; doi:10.3389/fphar.2019.00269)
Supplement: Supplementary file 1 [file Data_Sheet_1.docx]

**SUPPLEMENTARY RESULTS**

**CARDIOPROTECTIVE ACTIONS OF THE ANNEXIN-A1 N-TERMINAL PEPTIDE, Ac_2-26_, AGAINST MYOCARDIAL INFARCTION**

Chengxue Qin^^1,2,3^, Sarah Rosli^1^, Minh Deo^1^, Nga Cao^1^, Jesse Walsh^1^, Mitchel Tate^1,3^, Amy E Alexander^1^, Daniel Donner^1^, Duncan Horlock^1^, Renming Li^1^, Helen Kiriazis^1^, Man K S Lee^1^, Jane E Bourke^5^, Yuan Yang^4^, Andrew J Murphy^1^, Xiao-Jun Du^1^, Xiaoming Gao^1^, Rebecca H. Ritchie^1,2,3^^

^1^Baker Heart and Diabetes Institute, Melbourne 3004, ^2^Dept of Pharmacology and Therapeutics, University of Melbourne, Parkville 3010, ^3^Dept of Diabetes, Central Clinical School, Monash University, Melbourne 3004, ^4^Centre for Inflammatory Diseases, Monash University, Clayton 3168, ^5^Dept of Pharmacology, Monash University, Clayton 3168, Australia

^CXQ, RHR are joint corresponding authors.

**Running title:** Annexin-A1 cardioprotection

*Special issue of Resolution Pharmacology - innovative therapeutic approaches based on the biology of resolution to control chronic diseases of western societies*

**CORRESPONDENCE:**

Dr Cheng Xue Qin, PhD, Heart Failure Pharmacology, Baker Heart and Diabetes Institute, 75 Commercial Rd, Melbourne 3004 Australia. Adjunct Research Fellow (University of Melbourne). Phone+613-8532-1374; Fax+613-8532-1100; Email [chengxuehelena.qin@baker.edu.au](mailto:chengxuehelena.qin@baker.edu.au)

Prof Rebecca H. Ritchie, PhD, FCSANZ, FAHA; NHMRC Senior Research Fellow, Head Heart Failure Pharmacology, Baker Heart and Diabetes Institute, 75 Commercial Rd, Melbourne 3004 Australia. Phone+613-8532-1392; Fax+613-8532-1100; Email [rebecca.ritchie@baker.edu.au](mailto:rebecca.ritchie@baker.edu.au)

**Supplementary Tables**

**Supplementary Table S1:** Cell injury responses to Ac_2-26_ at concentrations 0.3-3 µM against hypoxia-reoxygenation (H-R) injury in neonatal cardiomyocytes *in vitro*, assessed by measuring troponin I release (ng/mL), whether present for the full duration of H-R (n=3 cardiomyocyte preparations) or added at the start of reoxygenation (n=4 preparations). *p<0.05 versus paired untreated control.

|  | **cTnI release (ng/mL)** | **cTnI release (ng/mL)** |
| --- | --- | --- |
|  | Ac_2-26_ present during H-R | Ac_2-26_ added at reoxygenation |
| Normoxia | 0.96±0.29 | 0.56±0.38 |
| H-R | 2.39±0.83 | 4.11±1.21 |
| H-R + 0.3 µM Ac_2-26_ | 2.65±1.21 | 2.28±0.80 |
| H-R + 1 µM Ac_2-26_ | 1.25±0.40* | 1.16±0.09* |
| H-R + 3 µM Ac_2-26_ | 2.33±1.22 | 1.91±1.10 |

**Supplementary Table S2:** Impact of either vehicle or Ac_2-26_ treatment on MI injury, as detected on cardiac gene expression relative to housekeeping gene 18s, 28 days after ischemic insult. #P<0.05, ##p<0.01 vs sham, and *P<0.05 vs vehicle-treated mice. One-way ANOVA with Dunnett’s *post-hoc* test. *post-hoc* test. Data are presented as mean±SEM, n indicates number of mice at end point.

|  | **Sham** | **MI + veh** | **MI + Ac_2-26_** |
| --- | --- | --- | --- |
| **n** | 7 | 5 | 7 |
| **Fibrotic markers** | | | |
| *mCtgf* | 1.0±0.4 | 10.0±4.3^#^ | 6.4±3.5 |
| *mTgf-β* | 1.0±0.1 | 1.3±0.2 | 0.8±0.2* |
| **Macrophage markers** | | | |
| *mCd68* | 1.0±0.5 | 4.8±2.6 | 5.9±3.7 |
| **M1-like macrophages** | | | |
| *mS100a8* | 1.0±0.7 | 10.2±6.6 | 13.6±8.5 |
| *mS100a9* | 1.0±0.2 | 7.4±2.4^##^ | 3.4±1.1 |
| **M2-like macrophages** | | | |
| *mArg-1* | 1.0±0.1 | 1.3±0.2 | 0.8±0.1 |
| *mCd206* | 1.0±0.1 | 0.8±0.4 | 0.3±0.1 |

**Supplementary Figure Legends**

**Supplementary Figure 1. Flow diagram showing mouse allocation and fate in this study.**

**Supplementary Figure 2. Impact of Ac_2-26_ on survival after MI.** Day 0 represents surgery day. Peak cardiac rupture tended to occur between days 4-5 post MI. Cohort 4, after 4 weeks MI. Log rank Mantel-COX comparison test (p=NS between groups). MI, myocardial infarction.


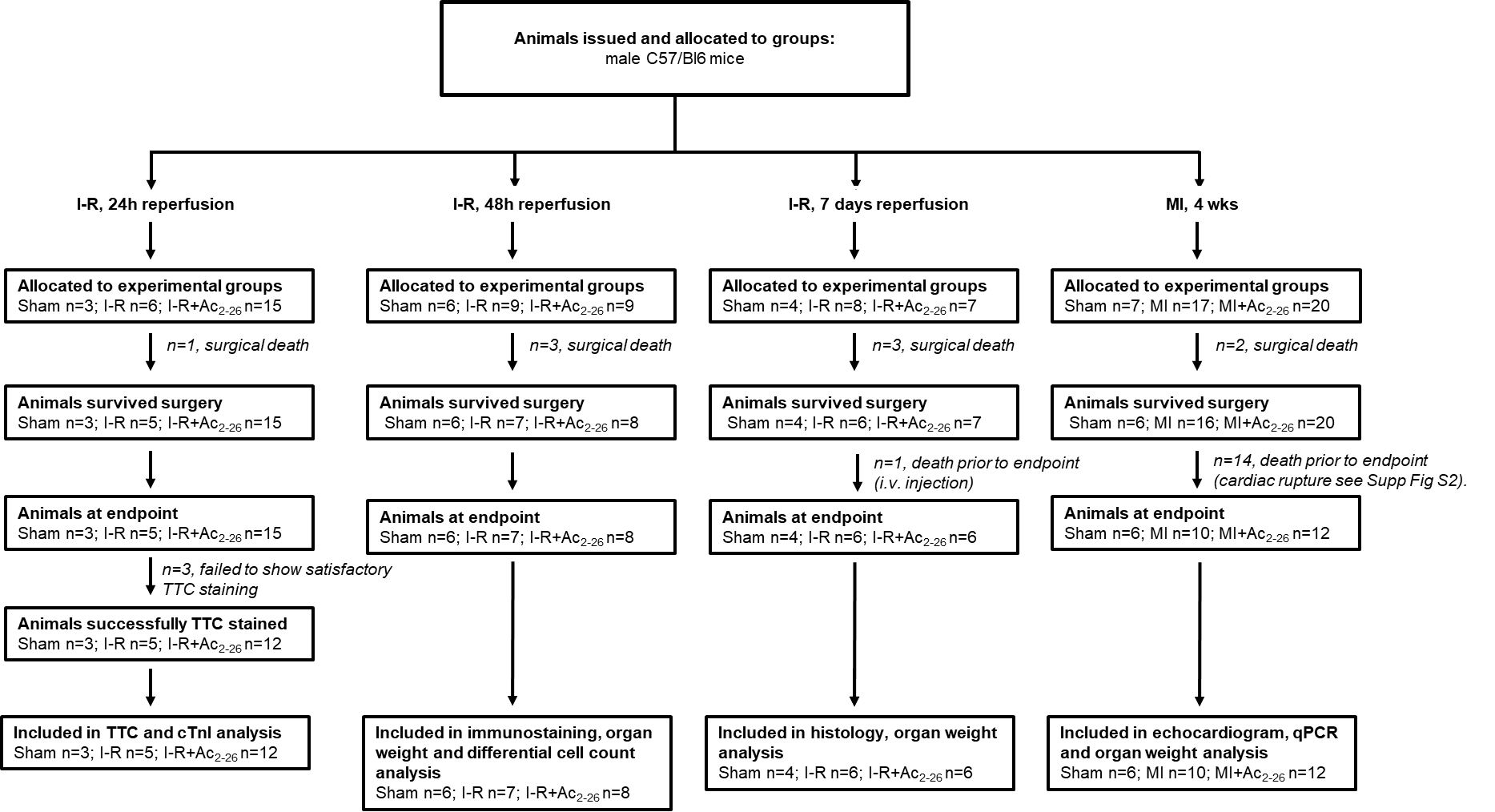


**Supplementary Figure 1.**

**Supplementary Figure 2**
